# High speed BLASTN: an accelerated MegaBLAST search tool

**DOI:** 10.1093/nar/gkv784

**Published:** 2015-08-06

**Authors:** Ying Chen, Weicai Ye, Yongdong Zhang, Yuesheng Xu

**Affiliations:** 1 Guangdong Province Key Laboratory of Computational Science, School of Mathematics and Computational Science, Sun Yat-sen University, Guangzhou 510275, P. R. China; 2 Department of Mathematics, Syracuse University, Syracuse, NY 13244, USA

## Abstract

Sequence alignment is a long standing problem in bioinformatics. The Basic Local Alignment Search Tool (BLAST) is one of the most popular and fundamental alignment tools. The explosive growth of biological sequences calls for speedup of sequence alignment tools such as BLAST. To this end, we develop high speed BLASTN (HS-BLASTN), a parallel and fast nucleotide database search tool that accelerates MegaBLAST—the default module of NCBI-BLASTN. HS-BLASTN builds a new lookup table using the FMD-index of the database and employs an accurate and effective seeding method to find short stretches of identities (called seeds) between the query and the database. HS-BLASTN produces the same alignment results as MegaBLAST and its computational speed is much faster than MegaBLAST. Specifically, our experiments conducted on a 12-core server show that HS-BLASTN can be 22 times faster than MegaBLAST and exhibits better parallel performance than MegaBLAST. HS-BLASTN is written in C++ and the related source code is available at https://github.com/chenying2016/queries under the GPLv3 license.

## INTRODUCTION

Identifying sequences (in a target database) having statistically significant local alignments with a given query is routine in computational biology. BLAST (1,2), a heuristic search tool developed for this purpose, has found substantial applications in protein science (3) such as secondary and tertiary structure prediction (4), functional annotation (5) and orthology mapping (6), and in nucleotide science including human genome variation detection (7) and gene prediction (8). BLAST builds a lookup table for the query, and scans the database for seeds, which are heuristic points for significant local alignments. These seeds are then extended to longer ungapped alignments and finally to gapped alignments.

Searching homologous sequences in a target database is a bottleneck in bioinformatics due to the exponential growth in the number of biological sequences (3). As a result, the acceleration of BLAST is an important problem. Over the past years, many methods were proposed to address this issue. They can be divided into two categories: hardware acceleration and improved indexing.

Hardware methods may either utilize parallel computing (9,10) or use custom-designed hardware such as field-programmable gate arrays (FPGAs) (11,12) and graphic processing units (GPUs) (13,14) to increase speed.^10^

The second category, improved indexing, creates an index for the database (15), instead of indexing the *query* as in BLAST. Widely used software packages belonging to this category include SSAHA (16), miBLAST (17), BLAT (18), indexed MegaBLAST (19), usearch (20) and DIAMOND (21).

MegaBLAST is currently the default module called from the program NCBI-BLASTN, which is a local nucleotide database search tool from the NCBI BLAST software distribution. Among the many solutions mentioned above, indexed MegaBLAST (19) and G-BLASTN (14) are dedicated to accelerating MegaBLAST and producing the same alignment results as MegaBLAST. Indexed MegaBLAST accelerates MegaBLAST by building an index for the target database. It stores the locations of each *k*-mer ($w$ ≥ *k*) that ends at every *s*-th (*s* = $w$ − *k* + 1) position in the database (19). The seed search algorithm first identifies *k*-seeds by using the index, and then checks if each *k*-seed is contained in a $w$-seed. The experiments conducted in (19) showed that it is 2–4 times faster than MegaBLAST. However, the checking procedure can be time-consuming when the queries are long and the database is large. The other search tool, G-BLASTN is an open-source GPU alternative. It utilizes GPUs in parallelizing the scanning stage of NCBI-BLASTN. Compared with the sequential MegaBLAST, G-BLASTN is 14.8 times faster.

The goal of this paper is to introduce HS-BLASTN, a parallel nucleotide sequence alignment tool that produces the same outputs as MegaBLAST with much faster computing speed. Formally, HS-BLASTN is a Burrow-Wheeler Transform (BWT) based solution to sequence alignment (see (22) for the definition of BWT). Over the past years, the BWT has been used intensively in next-generation sequencing (NGS) analysis. Many packages, such as Bowtie2 (23), BWA (24), cushaw2 (25), use the BWT as an indexing technique to map the NGS sequences (always contained in FASTQ format files) to the reference genomes. We adopt this indexing technique for the field of genomic database search—identifying all the statistically significant local alignments between a nucleotide query and a nucleotide database. Precisely, we use this data structure to accelerate MegaBLAST and produce the identical results. To do this HS-BLASTN employs a new database-derived lookup table based on the FMD-index introduced recently in (26), and uses an accurate and effective seeding method. This seeding method finds all seeds identified by MegaBLAST. HS-BLASTN is especially suitable for aligning a huge number of queries against a large database. Our experiments on searching two large query sets against the human genomic database show that HS-BLASTN achieves a significant speedup over MegaBLAST and much better parallel performance.

In this paper, we first review the procedure of MegaBLAST and the definitions of the FMD-index, and then describe the implementation of HS-BLASTN, including the lookup table, the seeding algorithm and the ungapped extension method. Finally, we compare the performances of HS-BLASTN and MegaBLAST by conducting experiments.

## MATERIALS AND METHODS

The MegaBLAST search procedure consists of three stages: the setup stage, the preliminary search stage and the traceback stage. At the setup stage, MegaBLAST prepares the options, queries and database, and then builds the lookup table for the queries. At the preliminary search stage, it scans each subject, finds seeds using the lookup table and performs a gap-free alignment algorithm on these seeds. The gap-free alignments exceeding a threshold score will trigger a gapped alignment. Those gapped alignments, whose scores exceed another threshold, will be saved as preliminary matches. Finally, the traceback stage considers the ambiguous nucleotides from the preliminary matches and returns them with the traceback information added. The traceback information includes the number and positions of insertions, deletions and matching letters.

The lookup table in MegaBLAST is a hash table. Each entry of the lookup table is an offset list that stores for one *k*-mer the offsets from the queries where the *k*-mer occurs. Since each letter is taken from {*A*, *C*, *G*, *T*}, the lookup table has 4^*k*^ entries. In the second stage, MegaBLAST walks through each subject to find $w$-seeds. Here it scans each of the subject's *k*-mer, calculates the *k*-mer's hash value, queries the lookup table and fetches the corresponding offset list. Each offset in the list yields a *k*-seed, which is a match of *k* consecutive nucleotides between the query and the subject. If $w$ is larger than *k*, we must also determine if this *k*-seed is contained in a $w$-seed. In that case, MegaBLAST scans the database in strides. The maximum stride that ensures that we can find all seeds is *s* = $w$ − *k* + 1. See (27,19,14) for more details.

The execution time of BLAST scales linearly in the size of the target sequence dataset. However, the sequence datasets grow exponentially making the acceleration of BLAST a pressing issue.

Many profiling studies (13,14) have revealed that the preliminary stage is the most computationally intensive stage among the three stages in BLAST and therefore the most promising place to accelerate BLAST. As mentioned in the ‘Introduction’ section, there are currently two major approaches in the literature to achieve this goal, one of which is to use different kinds of custom-designed hardware. For example, GPU-BLAST (13) (resp. G-BLASTN (14)) uses GPUs to parallelize the preliminary stage of BLASTP (1,2) (resp. BLASTN (28)). Another way, suggested by many studies (15), is to replace the query-index used in BLAST by a database-derived index. There are several software packages that employ such an index. However, these packages are either less sensitive than BLAST, due to *k*-mers missing from the index, or suffer from poor performance on long queries and large databases due to their indexing and seeding methods.

The key idea underlying HS-BLASTN is to replace the seeding step of MegaBLAST with a new lookup table and seeding algorithm. To this end, we build an FMD-index of the genomic database. We note that searching the database using its FMD-index gives us a bi-interval for each *k*-mer (see (26) for its definition). Thus, using the FMD-index we build a new lookup table storing the bi-intervals of all the *k*-mers, which are then used by a new seeding algorithm to find seeds. There are two advantages of HS-BLASTN over the other packages mentioned above. First, HS-BLASTN finds all the $w$-seeds ($w$ ≥ *k*). Second, HS-BLASTN checks whether a set of *k*-seeds are contained in $w$-seeds all at the same time, which makes it faster than indexed MegaBLAST that checks only each *k*-seed one at a time. Finally, we note that HS-BLASTN utilizes the same ungapped and gapped extension algorithms as used in MegaBLAST.

In the remainder of this section, we introduce the notations to be used later, and then describe our database-derived lookup table, seeding algorithm and ungapped extension stage that are used in HS-BLASTN.

### Notations

We use the finite order set Σ = {$,*A*, *C*, *G*, *T*} with $<*A* < *C* < *G* < *T* to represent the alphabet of DNA sequences. The letter $ is a sentinel that is used to mark the end of a sequence. For convenience, we also treat $, *A*, *C*, *G*, *T* as integers 0, 1, 2, 3, 4, respectively. Let *S* be a sequence with length |*S*|. For 0 ≤ *i* ≤ *j* < |*S*|, we denote by *S*[*i*] the *i*th symbol in *S* and *S*[*i*, *j*] the substring of length (*j* − *i* + 1) starting at the *i*th-position and ending at the *j*th-position. We call *S*_*i*_ ≔ *S*[*i*, |*S*| − 1] the *i*th suffix of *S*. For two strings *P* and *W*, we write *PW* as their concatenation. We also define $\overline{S}$ as the reverse complement of *S*. In addition, given a symbol *a* ∈ Σ, we denote by $\overline{a}$ the complement of *a*. As defined in (29), a string terminated with $ is called a *text*.

A sequence in the database is called a *subject*. A *k-mer* is a subsequence of length *k*. A $w$*-hit*, or $w$*-seed*, between a query *Q* and a subject *S* is a triplet (*q*, *s*, $w$) such that *Q*[*q*, *q* + $w$ − 1] matches *S*[*s*, *s* + $w$ − 1]. When the value of $w$ is known from the context, we simply call a $w$-hit ($w$-seed) a hit (seed).

HS-BLASTN processes non-A/C/G/T symbols (also called ambiguous symbols) the same way as MegaBLAST. Ambiguous symbols in the subjects are replaced randomly by nucleotides when the FMD-index is constructed, and will be recovered in the traceback stage. During the search, a symbol from the subject may be aligned to an ambiguous symbol from the queries. This case will be simply treated as a mismatch. See the Supplementary File for more details about treating ambiguous symbols.

### The lookup table for database

In this subsection, we describe the lookup table in HS-BLASTN. Because our lookup table is built atop the FMD-index and relies on the concept of the bi-interval, we first introduce the FMD-index and the bi-interval. Here, *S* represents a given text, *n* ≔ |*S*| denotes its length and *P* represents a given string with length *m* ≔ |*P*|.

Given a text *S*, as defined in (26), the *FMD-index* of *S* is the *FM-index* built for $S\overline{S}$. The FM-index was introduced in (30) as a fast string matching tool. FM-index is a compressed representation of the suffix array, the BWT and the occurrence array built upon *S*. We discuss these concepts below.

The *suffix array* *SA* of *S* is an array of integers in the range [0, *n* − 1] specifying the lexicographic ordering of the *n* suffixes of *S*, that is, a permutation of the integers {0, 1, …, *n* − 1} such that *S*_*SA*[0]_ < *S*_*SA*[1]_ < ⋅⋅⋅ < *S*_*SA*[*n* − 1]_. The *suffix array interval* [*I*^*l*^(*P*), *I*^*u*^(*P*)] of a given string *P* in *S* is defined to be the interval in *SA* such that *P* is a prefix of *S*_*SA*[*k*]_ for all *I*^*l*^(*P*) ≤ *k* ≤ *I*^*u*^(*P*), but not a prefix of any other suffix of *S*. For convenience, we also denote by *I*^*s*^(*P*) the size of this interval, that is, *I*^*s*^(*P*) ≔ *I*^*u*^(*P*) − *I*^*l*^(*P*) + 1.

The *Burrows–Wheeler Transform*, or *BWT*, is a data compression technique introduced in (22). The BWT of *S* is a sequence *B* computed by *B*[*i*] = *S*[*SA*[*i*] − 1] for *SA*[*i*] > 0 and *B*[*i*] =$ otherwise. We need another two arrays *C*[*a*] and *O*[*a*][*i*] for any *a* ∈ Σ and 0 ≤ *i* < *n*, where *C*[*a*] stores the number of symbols in *S* that are strictly smaller than *a*, and *O*[*a*][*i*] is called the *occurrence array*, which is a two-dimensional array that stores the occurrence of *a* in *B*[0, *i*]—the substring of *B* that consists of the first *i* + 1 symbols of *B*.

We now introduce the bi-interval of *P* in *S*. Given the FMD-index of *S*, it holds that $I^s(P)=I^s(\overline{P})$ where we define the bi-interval of *P* in *S* as $\omega (P):=[I^l(P), I^l(\overline{P}),I^s(P)]$. Once we know the bi-interval of *P*, we can use the backward extension algorithm (Algorithm 2 in (26)) to get the bi-interval of *aP* and use the forward extension algorithm (Algorithm 3 in (26)) to get the bi-interval of *Pa*, for any *a* ∈ Σ.

We build a lookup table for the database based on the FMD-index. Our lookup table is also a hash table. Unlike the lookup tables used in MegaBLAST or in indexed MegaBLAST, each cell in our lookup table stores the bi-interval of one *k*-mer. The number of entries in the lookup table is 4^*k*^. Our lookup table has three advantages. One, we do not have to build the lookup table each time when searching against the database. Two, our lookup table occupies much less space than that in indexed MegaBLAST. Finally three, our lookup table is more effective than the ones used in MegaBLAST and indexed MegaBLAST, as showed by our experimental results presented in ‘Experimental Results’ section.

### The seed search algorithm

Our seed search algorithm will find all the $w$-seeds between the query and the database. It consists of two steps: bi-interval identification and occurrence position detection.

**Figure 1.**
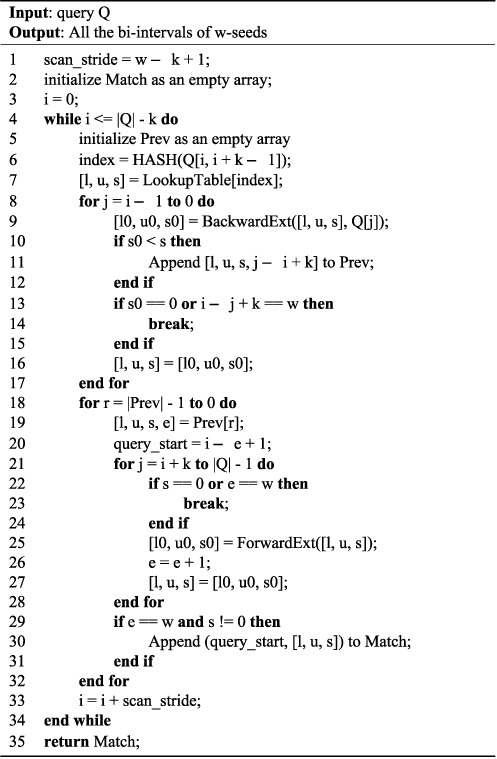
Bi-interval Identification: the first step of the seed search algorithm in HS-BLASTN.

The first step, which determines the bi-intervals of $w$-seeds, is described in Figure 1. Given a query to an HS-BLASTN search, we scan the query in strides (line 33). As in MegaBLAST, the maximum stride which ensures that all the $w$-seeds will be found is $w$ − *k* + 1 (line 1). For each *k*-word from the query encountered, we calculate its hash value (line 6) and fetch its bi-interval in the lookup table (line 7). For this bi-interval, we use the backward extension algorithm BackwardExt to conduct the backward search (line 9). If *I*^*s*^(*Q*[*j*, *i* + *k* − 1]) is smaller than *I*^*s*^(*Q*[*j* + 1, *i* + *k* − 1]) (line 10), then we know that some (*k* + *i* − *j*)-seeds cannot be extended in the left direction to yield (*k* + *i* − *j* + 1)-seeds. In this case, we extend it to the right, using the forward extension algorithm ForwardExt (line 25) and check if the bi-interval of *Q*[*j* + 1, *j* + $w$] is not empty (line 29).

**Figure 2.**
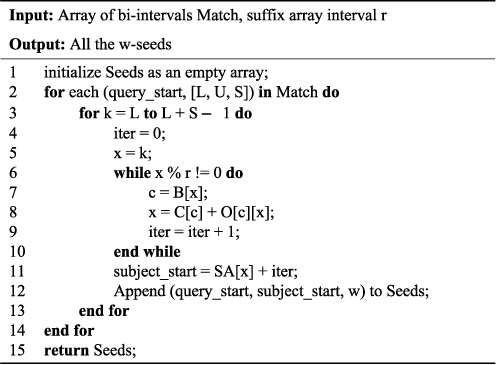
Occurrence Position Detection: the second step of the seed search algorithm in HS-BLASTN.

The second step is illustrated in Figure 2. It takes the bi-intervals produced by step 1 as input to determine the exact positions of all the occurrences of $w$-seeds in the database. To this end, for each bi-interval [*l*, *u*, *s*] and each *k* ∈ [*l*, *u*, *s*], we find *SA*[*k*]. In practice, the suffix array *SA* may be too large to reside in RAM. To overcome this difficulty, the implementation of the FMD-index in (26) stores explicitly only *SA*[0], *SA*[*r*], *SA*[2*r*], …, *SA*[|*S*|/*r* − 1], where *r* ≥ 1 is a fixed integer and is referred to as the *suffix array interval*. If *k* is a multiple of *r*, then *SA*[*k*] is available and it can be used immediately. Otherwise, we must iteratively call the *Last-to-First column mapping* (LF-mapping) (lines 7-8, see (30) for details) until we reach the position *x* that is divisible by *r* (line 6), and get *SA*[*k*] = *SA*[*x*] + *iter* (line 11), where *iter* is the number of iterations. The choice of the value of *r* is a tradeoff between efficiency and memory usage. In (26), *r* is set to be 32 so that the FMD-index built for the human genome database can reside in 8GB RAM.

We now analyze the time complexity of our seeding algorithm. The BackwardExt and ForwardExt are called $\mathcal {O}(|Q|)$ times in step 1, where |*Q*| denotes the length of query *Q*. A calling of BackwardExt or ForwardExt involves invoking the LF-mapping four times (see Algorithm 2 of (26)). Since the LF-mapping can be computed in constant time, step 1 can be finished in $\mathcal {O}(|Q|)$ time. In step 2, we denote the longest time needed to retrieve *SA*[*k*] by *t*_max _, which is also a constant. If the number of $w$-seeds is *s*, then step 2 can be conducted in $\mathcal {O}(st_{\max }) = \mathcal {O}(s)$ time.

### From seeds to ungapped alignments

Both HS-BLASTN and MegaBLAST use the same ungapped extension algorithm to extend a subset of the seeds to ungapped alignments. However, MegaBLAST finds seeds and performs ungapped alignments at the same time, while HS-BLASTN triggers ungapped alignments after all the seeds have been found. In what follows, we first review the scanning stage in MegaBLAST and then introduce the ungapped extension stage in HS-BLASTN. For this purpose, we need another two notions. Given a seed (*q*, *s*, $w$), the number *d* ≔ *s* − *q* is referred to as its *diagonal*. A seed is said to lie on diagonal *d* if its diagonal is *d*. If two seeds lie on the same diagonal, the one with a smaller subject offset will be extended first. The ungapped alignment of a seed can also be identified by a triplet $(q\_{\rm start}, s\_{\rm start}, L)$, where $q\_{\rm start}, s\_{\rm start}$ represent the start offsets in the query and in the subject, respectively, and *L* is the length of the alignment. The end subject offset, $s\_{\rm start} + L - 1$, of this alignment is called the *diagonal offset* produced by the seed.

At the preliminary search stage, MegaBLAST maintains a hash table. Each entry of the table corresponds to one diagonal and contains the diagonal offset produced by the last seed that lies on the diagonal. When MegaBLAST finds a seed, it calculates the seed's diagonal and fetches the corresponding diagonal offset from the hash table. If the diagonal offset exceeds the seed's subject offset, then no ungapped extension is necessary. Otherwise, an ungapped extension is performed on the seed and the diagonal offset in the corresponding hash cell will be replaced by the one produced by the seed.

Once HS-BLASTN finds all the seeds, it sorts them using the quick sort algorithm (31). After sorted, the seeds that lie on the same diagonal cluster together and those having smaller subject offsets lie on the left hand side of those having larger subject offsets. According to (31), the computing time required for the sort is $\mathcal {O}(s\log s)$, where *s* is the number of seeds. Our experiments show that the sort is very fast in practical computation. In fact, the sorting procedure occupies <1% of the total execution time.

For seeds that lie on the same diagonal, the ungapped extension process begins with the left most seed. At the same time, we keep track of a number *D*, where *D* is initialized to be the diagonal offset produced by the left most seed. If the next seed's subject offset is less than *D*, then we know that it can be discarded. Otherwise it will trigger an ungapped extension and *D* will be updated with the seed's diagonal offset.

## IMPLEMENTATION

We now describe the implementation of HS-BLASTN, including the implementation of the FMD-index, the parallel design and the usage of HS-BLASTN.

### The implementation of the FMD-index

We modify the implementation of the FMD-index in (26). The first modification is that the lookup table is built for the target database and is integrated into the FMD-index. The lookup table plays a crucial role in our seeding algorithm. The second is that the value of the suffix array interval *r* is changed from 32 in (26) to 8. When a query is long and the database is large, many bi-intervals of $w$-seeds will be obtained, which implies that the time-consuming LF-mapping will be repeatedly called many times. As a result, the performance of HS-BLASTN deteriorates. To settle this problem, a smaller value of *r* should be used.

**Figure 3.**
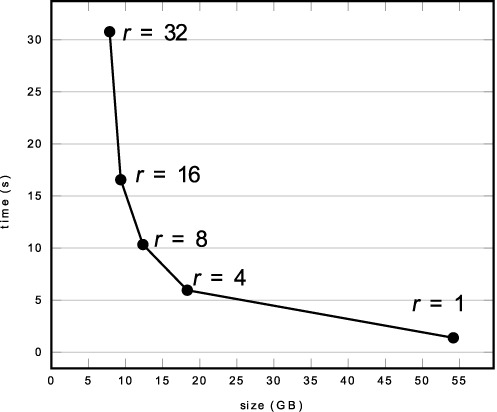
When searching against the human genome database, the running time of step 2 of the seeding algorithm and the size of the FMD-index under different values of *r*. One CPU thread is used. The choice of *r* is a tradeoff between efficiency and memory usage: the smaller value of *r*, the less execution time, and the larger value of *r*, the smaller size of the FMD-index.

It is a challenge to choose the value of *r*. There are two factors that must be considered: efficiency and memory usage. To pick up an appropriate *r*, we build several FMD-indices for the human genomic database. These indices are constructed with different values of *r*. We then search a set of queries (these queries consist of the first 200 000 queries of query set 0 and query set 1, details of query sets 0 and 1 can be found in ‘Experimental Results’ section) using these indices and examine the execution time of step 2 of the seeding algorithm (Figure 2). The execution time and the sizes of the indices are shown in Figure 3, From the figure we can see that, a small value of *r* yields high performance but requires large memory occupation, while a large value of *r* yields slow speed and needs small memory usage. We choose *r* = 8 due to the fact that under this value, HS-BLASTN is feasible to run on a computer equipped with 16GB RAM and is fast enough, as showed by the experiments.

### Parallel design

**Figure 4.**
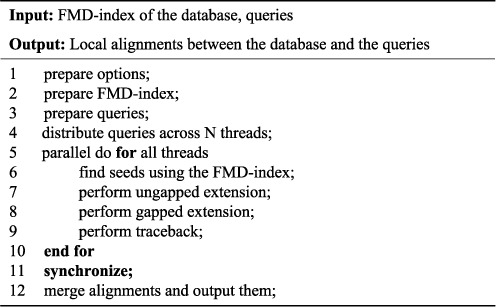
The whole HS-BLASTN search procedure.

To make full use of the computational power provided by multi-core computers, we parallelize HS-BLASTN by using multiple CPU threads. Users are allowed to specify a desired number of searching threads (option-num_threads, as in blastn). HS-BLASTN first prepares the database and the queries. Because the search (the preliminary search stage and the traceback stage) on different queries are independent, HS-BLASTN distributes the queries across the searching threads. Each thread launches the search engine concurrently. The searching threads synchronize with each other when they finish their tasks. Finally, HS-BLASTN merges the results from all the threads into a single output. The whole HS-BLASTN search with *N* threads proceeds as Figure 4.

During the search, the memory image of the FMD-index is shared by all threads. As a result, the memory footprint will not grow substantially when multiple threads are used.

### The usage of HS-BLASTN

Using HS-BLASTN to conduct a database search involves two steps. The first step is to build the FMD-index for the target database, using the index command. The command to build the FMD-index for database human.fa looks like the following.

$hs-blastn index human.fa

The second step is to use the align command to search against the target database. HS-BLASTN supports a frequently used subset of the options in blastn. For example, we use the following command line to run HS-BLASTN in our experiments.

$hs-blastn align -db <database> \

-query <file> -outfmt 7 -dust yes \

-num_threads <Integer> \

-window_masker_db <masker_db>

## EXPERIMENTAL RESULTS

To assess the execution time of HS-BLASTN and MegaBLAST we run HS-BLASTN and MegaBLAST on two query sets under different numbers of CPU threads. We will also compare their parallel performance when multiple CPU threads are used. The output results of the two alignment tools are the same in the two query sets. We first introduce the general setup, datasets and test methods, and then discuss the experimental results.

Due to the large size of the FMD-index, before lunching the search engine, HS-BLASTN spends some time (about 6 s on our server) to load the index into RAM (step 2 of Figure 4). However, because the index is loaded only once, there is an overall performance benefit when aligning a large number of queries. As a result, compared to the long searching time, the time spent on loading the index can be almost neglected. Hence, to demonstrate the performance advantage of HS-BLASTN over MegaBLAST, we compare their execution time of searching two very large query sets.

### General setup and datasets

The experiments are all conducted on a Linux server with two six-core Intel Xeon E5-2620 CPUs and has more than 16GB of RAM. The MegaBLAST that we use is the 64-bit build and is built from the source code of BLAST version 2.2.30+. Both the source codes of BLAST and HS-BLASTN are compiled with GCC version 4.4.6. The code of HS-BLASTN is compiled with the -O3 level of optimization set. The command lines used for building BLAST and HS-BLASTN are given in the Supplementary File.

#### Database

The database that we choose is the human build 38 (http://hgdownload.soe.ucsc.edu/downloads.html#human). We mask the database with the WindowMasker (32). The size of the FMD-index built for the database is about 12.2GB.

#### Queries

The queries are all Homo Sapience sequences. We extract two query sets from the file Hs.seq.all (ftp://ftp.ncbi.nih.gov/repository/UniGene/). Query set 0 consists of 2 millions of short queries with length ranging from 100 to 500. Query set 1 consists of about 870 000 long queries with length being 800–4000.

### Test methods

We run both HS-BLASTN and MegaBLAST on each query set in the batch mode under different numbers of CPU threads. That is, each command line call of HS-BLASTN or MegaBLAST takes a query set as input. Our test method is different from that in (19,14), in which each command line call handles only one query at a time. Before the experiments are conducted, the FMD-index, which is constructed only once, has been built (the index command). The time spent on building the FMD-index is not included in the HS-BLASTN running time. The running time of HS-BLASTN and MegaBLAST that we record is the wall clock time and is measured by the standard time utility. We run each test five times and report the average execution time.

### Results

In this subsection, we compare the performance of HS-BLASTN with that of MegaBLAST on each query set under different numbers of CPU threads. To this end, we use *TH*(*q*, *n*) (resp. *TM*(*q*, *n*)) to represent the execution time of HS-BLASTN (resp. MegaBLAST) running on query set *q* under *n* CPU threads. We define,

(1) \begin{equation*} S(q,n):=\frac{TM(q,n)}{TH(q,n)} \end{equation*}

as the relative speedup achieved by HS-BLASTN in comparison to MegaBLAST when both alignment tools running on query set *q* under *n* CPU threads.

**Figure 5.**
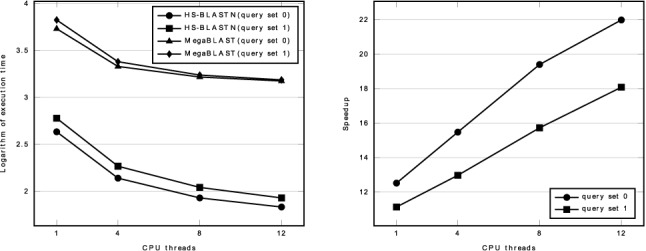
Logarithm of the execution time and speedup on searching against the human genomic database. **Left:** Each curve shows on a specific query set the logarithm of the execution time of HS-BLASTN or MegaBLAST as a function of CPU threads. The execution time of HS-BLASTN in each test includes about 6 s that are used for loading the FMD-index into RAM. **Right:** Each curve represents the speedup *S*(*q*, *n*) (see Equation (1)) achieved by HS-BLASTN in comparison to MegaBLAST when both alignment tools run on the same query set under the same number of CPU threads. On each query set, the execution time decreases when the number of CPU threads is raised. When both are running under 12 CPU threads, HS-BLASTN is 22 and 18 times faster than MegaBLAST on query set 0 and query set 1, respectively. On each query set, as we raise the number of CPU threads, the execution time of HS-BLASTN drops faster than that of MegaBLAST, which indicates improved performance with increasing thread number.

The experimental results are depicted in Figure 5. The running time of HS-BLASTN is close to 0 in comparison with that of MegaBLAST. For the sake of comparison, we demonstrate the logarithm of the execution time instead. The execution time can be found in the Supplementary File. Figure 5 also shows the speedup *S*(*q*, *n*). In each test, HS-BLASTN loads the FMD-index into RAM before running the searching procedure. On our server, this overhead costs about 6 s, which are included in the execution time of HS-BLASTN.

HS-BLASTN is faster than MegaBLAST in all the tests, especially on query set 0. When both running under 12 CPU threads, HS-BLASTN is 22 and 18 times faster than MegaBLAST on query set 0 and query set 1, respectively. The speedup on query set 0 is larger than that on query set 1 under all the CPU threads. This is because on short queries, the seed identification step in MegaBLAST consumes a larger percentage of the total execution time.

HS-BLASTN exhibits much better parallel performance than MegaBLAST. Although the execution time of the two aligners on all query sets decreases as we raise the number of CPU threads, HS-BLASTN scales better than MegaBLAST. The execution time of HS-BLASTN decreases more rapidly than that of MegaBLAST and the speedup *S*(*q*, *n*) becomes larger, which can be seen from Figure 5.

## CONCLUSIONS

We have introduced HS-BLASTN, a nucleotide database search tool that shows a computational speed improvement compared to MegaBLAST. HS-BLASTN accelerates MegaBLAST by creating a lookup table that stores the bi-intervals of all the *k*-mers and uses a seeding method that scans the queries in strides. We have demonstrated by experiments that HS-BLASTN exhibits a great performance advantage over MegaBLAST and a much better parallel performance than MegaBLAST. Because the number of biological sequences grows exponentially, HS-BLASTN, with its improved execution speed (10–20×) over MegaBLAST, is an important advance for bioinformatics genomic search.

## SUPPLEMENTARY DATA

Supplementary Data are available at NAR Online.

SUPPLEMENTARY DATA

## References

[1] Altschul S.F., Gish W., Miller W., Myers E.W., Lipman D.J. (1990). Basic local alignment search tool. J. Mol. Biol..

[2] Altschul S.F., Madden T.L., Schäffer A.A., Zhang J., Zhang Z., Miller W., Lipman D.J. (1997). Gapped BLAST and PSI-BLAST: a new generation of protein database search programs. Nucleic Acids Res..

[3] Daniels N.M., Gallant A., Peng J., Cowen L.J., Baym M., Berger B. (2013). Compressive genomics for protein databases. Bioinformatics.

[4] Rost B., Yachdav G., Liu J. (2004). The predictprotein server. Nucleic Acids Res..

[5] Loewenstein Y., Raimondo D., Redfern O.C., Watson J., Frishman D., Linial M., Orengo C., Thornton J., Tramontano A. (2009). Protein function annotation by homology-based inference. Genome Biol..

[6] Tatusov R.L., Galperin M.Y., Natale D.A., Koonin E.V. (2000). The COG database: a tool for genome-scale analysis of protein functions and evolution. Nucleic Acids Res..

[7] Sachidanandam R., Weissman D., Schmidt S.C., Kakol J.M., Stein L.D., Marth G., Sherry S., Mullikin J.C., Mortimore B.J., Willey D.L. (2001). A map of human genome sequence variation containing 1.42 million single nucleotide polymorphisms. Nature.

[8] Allen J.E., Pertea M., Salzberg S.L. (2004). Computational gene prediction using multiple sources of evidence. Genome Res..

[9] Oehmen C., Nieplocha J. (2006). ScalaBLAST: a scalable implementation of BLAST for high-performance data-intensive bioinformatics analysis. IEEE Trans. Parallel Distrib. Syst..

[10] Oehmen C.S., Baxter D.J. (2013). ScalaBLAST 2.0: rapid and robust BLAST calculations on multiprocessor systems. Bioinformatics.

[11] Herbordt M.C., Model J., Sukhwani B., Gu Y., VanCourt T. (2007). Single pass streaming BLAST on FPGAs. Parallel Comput..

[12] Sotiriades E., Dollas A. (2007). A general reconfigurable architecture for the BLAST algorithm. J. VLSI Signal Process..

[13] Vouzis P.D., Sahinidis N.V. (2011). GPU-BLAST: using graphics processors to accelerate protein sequence alignment. Bioinformatics.

[14] Zhao K., Chu X. (2014). G-BLASTN: accelerating nucleotide alignment by graphics processors. Bioinformatics.

[15] Jiang X., Zhang P., Liu X., Yau S.S.-T. (2007). Survey on index based homology search algorithms. J. Supercomput..

[16] Ning Z., Cox A.J., Mullikin J.C. (2001). SSAHA: a fast search method for large DNA databases. Genome Res..

[17] Kim Y.J., Boyd A., Athey B.D., Patel J.M. (2005). miBLAST: scalable evaluation of a batch of nucleotide sequence queries with BLAST. Nucleic Acids Res..

[18] Kent W.J. (2002). BLAT-the BLAST-like alignment tool. Genome Res..

[19] Morgulis A., Coulouris G., Raytselis Y., Madden T.L., Agarwala R., Schäffer A.A. (2008). Database indexing for production MegaBLAST searches. Bioinformatics.

[20] Edgar R.C. (2010). Search and clustering orders of magnitude faster than BLAST. Bioinformatics.

[21] Buchfink B., Xie C., Huson D.H. (2015). Fast and sensitive protein alignment using DIAMOND. Nat. Methods.

[22] Burrows M., Wheeler D.J. (1994). A block-sorting lossless data compression algorithm.

[23] Langmead B., Salzberg S.L. (2012). Fast gapped-read alignment with Bowtie 2. Nat. Methods.

[24] Li H., Durbin R. (2009). Fast and accurate short read alignment with Burrows–Wheeler transform. Bioinformatics.

[25] Liu Y., Schmidt B. (2012). Long read alignment based on maximal exact match seeds. Bioinformatics.

[26] Li H. (2012). Exploring single-sample SNP and INDEL calling with whole-genome de novo assembly. Bioinformatics.

[27] Camacho C., Coulouris G., Avagyan V., Ma N., Papadopoulos J., Bealer K., Madden T.L. (2009). BLAST+: architecture and applications. BMC Bioinformatics.

[28] Zhang Z., Schwartz S., Wagner L., Miller W. (2000). A greedy algorithm for aligning DNA sequences. J. Comp. Biol..

[29] Sirén J. (2009). Compressed suffix arrays for massive data. Proceedings of the 16th International Symposium on String Processing and Information Retrieval.

[30] Ferragina P., Manzini G. (2000). Opportunistic data structures with applications. Proceedings of the 41st Annual Symposium on Foundations of Computer Science.

[31] Cormen T.H., Leiserson C.E., Rivest R.L., Stein C. (2009). Introduction to Algorithms.

[32] Morgulis A., Gertz E.M., Schäffer A.A., Agarwala R. (2006). WindowMasker: window-based masker for sequenced genomes. Bioinformatics.

