# Supplementary material for: High speed BLASTN: an accelerated MegaBLAST search tool
Source: Nucleic Acids Res. 2015 Aug 6;43(16):7762–8. doi: 10.1093/nar/gkv784 (PMC4652774; doi:10.1093/nar/gkv784)
Supplement: SUPPLEMENTARY DATA [file supp_43_16_7762__index.html]

High speed BLASTN: an accelerated MegaBLAST search tool — SUPPLEMENTARY DATA 

# High speed BLASTN: an accelerated MegaBLAST search tool

## SUPPLEMENTARY DATA

- SUPPLEMENTARY DATA
