# Supplementary material for: High speed BLASTN: an accelerated MegaBLAST search tool
Source: Nucleic Acids Res. 2015 Aug 6;43(16):7762–8. doi: 10.1093/nar/gkv784 (PMC4652774; doi:10.1093/nar/gkv784)
Supplement: SUPPLEMENTARY DATA [file supp_gkv784_nar-00226-z-2015-File004.pdf]

Contents

1. The treatment of ambiguous letters 1
2. Experiment design 2
2.1 Download and install BLAST 2
2.2 Download and install HS-BLASTN 2
2.3 Download the queries 2
2.4 Preparing the database 3
2.5 Building the database for MegaBLAST 3
2.6 Building the FMD-index for HS-BLASTN 3
2.7 Commandline to run MegaBLAST 4
2.8 Commandline to run HS-BLASTN 4
3. Execution time 4

1. The treatment of ambiguous letters

BLASTN employs two sequence encoding schemes: the ncbi2na scheme and the blastna scheme. In ncbi2na scheme, the letters A, C, G, T will be encoded as integers 0, 1, 2, 3, respectively. Each ambiguous character is randomly assigned one of the nucleic acids. In blastna scheme, the ambiguous letters are encoded separately, as shown in the following table:

Nucleotide	value	nucleotide	value
A	0	W	8
C	1	S	9
G	2	B	10
T	3	D	11
R	4	H	12
Y	5	V	13
M	6	N	14
K	7	-	15

In BLASTN, the queries and the subjects are encoded differently. The queries are encoded using the blastna scheme throughout the BLASTN search. While the subjects are encoded in ncbi2na format in the preliminary search (including the seeding step, the ungapped extension step and the score-only gapped extension step), in blastna format in the traceback search (the traceback step). See The Developer's Guide to BLAST for more details.

HS-BLASTN encodes the queries and the subjects in the same way as BLASTN. Since the FMD-index is only used for finding seeds, each ambiguous character in the database is randomly replaced by one of the nucleic acids when building the FMD-index.

2. Experiment design

To conduct the following experiments, a computer with 16GB RAM and runs the 64-bit linux operating system is required.

2.1 Download and install BLAST

The BLAST that we use in the experiments is built from the source code ncbi-blast-2.2.30+-src.tar.gz. We use the following commands for building BLAST:

```
tar xzvf ncbi-blast-2.2.30+-src.tar.gz
cd ncbi-blast-2.2.30+-src/c++/
./configure --with-mt --with-optimization --without-debug –with-64
make
```

The following three tools: blastn, makeblastdb, and windowmasker, can be found in the directory ncbi-blast-2.2.30+-src/c++/ReleaseMT/bin. These three tools, together with hs-blastn, the database and the queries, should be put in the same directory.

2.2 Download and install HS-BLASTN

```
git clone https://github.com/chenying2016/queries.git
cd queries/hs-blastn-0.0.3+-src
make
```

2.3 Download the queries

The queries have been uploaded to

https://github.com/chenying2016/experimental_queries

We should download the queries by clicking the "Download ZIP" button at the bottom right corner of the website:

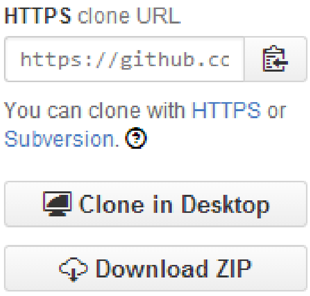

If we use the “git” to clone the queries, it will stay at “unpacking objects” for a long time. After downloading, we obtain a file experimental_queries-master.zip. We then use the following commands to decompress the queries:

```
unzip experimental_queries-master.zip
cd experimental_queries-master
cat experimental_queries.tar.bz2.a* | tar xjv
```

There are two FASTA files: HSQueriesSmall.fa and HSQueriesLarge.fa. HSQueriesSmall.fa consists of 2,000,000 short queries whose length is ranging from 100 to 500. HSQueriesLarge.fa consists of about 870,000 long queries. The length is from 800 to 40000.

2.4 Preparing the database

Download the human genomic database from

http://hgdownload.cse.ucsc.edu/goldenPath/hg38/bigZips/hg38.fa.gz.

Unpack the file and put all the sequences into one FASTA formated file with name hg38.fa.

2.5 Building the database for MegaBLAST

Build a blastdb:

```
./makeblastdb -in hg38.fa -input_type fasta -dbtype nucl
```

Generate a frequency counts file for the database using the WindowMasker:

```
./windowmasker -in hg38.fa -infmt blastdb -mk_counts -out hg38.fa.counts
```

Convert the frequency file from text format to obinary format:

```
./windowmasker -in hg38.fa.counts -sformat obinary -out hg38.fa.counts.obinary -convert
```

2.6 Building the FMD-index for HS-BLASTN

```
./hs-blastn index hg38.fa
```

This command will generate the FMD-index for hg38.fa, which consists of 4 files:

1) hg38.fa.header The header file, contains the header information of each subject.
2) hg38.fa.bwt This file contains the bwt.
3) hg38.fa.sa This file contains the suffix array.
4) hg38.fa.sequence All the subjects will be concatenated into this file (the headers are excluded), encoded in blastna format.

2.7 Commandline to run MegaBLAST

```
time ./blastn -task megablast -db hg38.fa -query HSQueriesSmall.fa -out
results_HSQueriesSmall.fa -outfmt 7 -window_masker_db hg38.fa.counts.obinary -num_threads 1
```

The above command line takes HSQueriesSmall.fa as query input. The results are written to file results_HSQueriesSmall.fa with format 7. It runs with 1 CPU thread. The system tool time is used to measure the execution time and the real time will be reported.

2.8 Commandline to run HS-BLASTN

```
time ./hs-blastn align -db hg38.fa -window_masker_db hg38.fa.counts.obinary -query
HSQueriesSmall.fa -out results_HSQueriesSmall.fa -outfmt 7 -num_threads 1
```

3. Execution time

We list the wall clock time (seconds) of HS-BLASTN and MegaBLAST on the two query sets under different CPU threads. In HS-BLASTN, each execution time includes about 6 seconds for pre-loading the FMD-index. Each test is run 5 times and the average execution time is reported.

Program	HS-BLASTN				MegaBLAST			
Cpu threads	1	4	8	12	1	4	8	12
HSQueriesSmall	430	138	85	68	5384	2136	1649	1495
HSQueriesLarge	600	185	110	85	6680	2400	1730	1537
